# P-484. Community Food Pantries Provide Unique Opportunities for Linkage to HIV Care in Newly Diagnosed Individuals

**DOI:** 10.1093/ofid/ofae631.683

**Published:** 2025-01-29

**Authors:** Marc Pickard, Corey Rosmarin-DeStefano, Christian Mendez-Baez, Karina Landaverde, Keven Tirado, Flavia Escalante, Sonia Vera, Mauricio Caro, Diana Finkel

**Affiliations:** New Jersey Medical School, Rutgers University, Newark, New Jersey; North Jersey Community Research Initiative, Newark, New Jersey; North Jersey Community Research Initiative, Newark, New Jersey; North Jersey Community Research Initiative, Newark, New Jersey; North Jersey Community Research Initiative, Newark, New Jersey; North Jersey Community Research Initiative, Newark, New Jersey; North Jersey Community Research Initiative, Newark, New Jersey; North Jersey Community Research Initiative, Newark, New Jersey; Rutgers NJMS, Newark, New Jersey

## Abstract

**Background:**

Food insecurity has been associated with sexual risk behavior that increases Human Immunodeficiency Virus (HIV) risk in cis women and sexual and gender minority (SGM) individuals such as transactional sex for money or food and multiple sexual partners. SGM people and cis women experiencing insecure immigration status also often face financial and housing instability, stigma, discrimination, lack of social support as well as food insecurity. North Jersey Community Research Initiative (NJCRI) is a non- profit organization in Newark, NJ that provides medical care and support services for highly vulnerable and hard to reach patient populations including unhoused individuals, people living with HIV/AIDS, LGBQTIA community members, people who use drugs and new immigrants. In 2023, NJCRI expanded food pantry status neutral services to the community and offered onsite HIV counselling and testing to pantry visitors.

**Methods:**

From April 2023 to April 2024, NJCRI provided HIV testing for 3630 individuals with 1025 being tested at the pantry.

**Results:**

There were 63 total positive tests with 13 diagnosed at the pantry. Of those diagnosed at the pantry, 2/3 (9) identified as SGM and 1/3 (4) as cis women. All the SGM individuals were Latinx. All reported to be new migrants. All diagnosed were linked to care and services and started on ART on the day of diagnosis.

**Table:** 

Organization	Total Test (Statewide)	NJCRI Pantry Total	Total (+) - Statewide	NJCRI Pantry - (+)
NJCRI	3630	1025	63	13
**Self-Identification**				
SGM	n/a	x	42	9
Cis-Women	n/a	x	9	4
**Ethnicity**				
Latinx	n/a	n/a	42	13
Black	n/a	n/a	17	0
Other	n/a	n/a	4	0

**Conclusion:**

Migrant individuals from known groups for higher HIV associated risk experiencing food insecurity benefit from HIV testing at nontraditional testing sites. Food pantries run by trusted community partners who provide integrated HIV care and prevention services can serve as entry points for HIV care for new immigrants. Community organizations serving this community should be aware of the opportunity to engage individuals in HIV care and prevention services and link to care.

**Disclosures:**

**All Authors**: No reported disclosures

